# Does ( −)-epigallocatechin-3-gallate protect the neurotoxicity induced by bisphenol A in vivo?

**DOI:** 10.1007/s11356-021-18408-z

**Published:** 2022-01-11

**Authors:** Manar Mohammed El Tabaa, Samia Salem Sokkar, Ehab Sayed Ramdan, Inas Zakria Abd El Salam, Anis Anis

**Affiliations:** 1 Pharmacology & Environmental Toxicology, Environmental Studies & Research Institute, University of Sadat City, Sadat, Egypt; 2 Pharmacology & Toxicology, Faculty of Pharmacy, Tanta University, Tanta, Egypt; 3 Psychiatry, Faculty of Medicine, Tanta University, Tanta, Egypt; 4 Medicinal Plants, Environmental Studies & Research Institute, University of Sadat City, Sadat, Egypt; 5 Pathology, Faculty of Veterinary Medicine, University of Sadat City, Sadat, Egypt

**Keywords:** Bisphenol A, ( −)-Epigallocatechin-3-gallate, Adiponectin, Uridine glucuronosyltransferases, Oxidative damage, Neurotoxicity

## Abstract

Bisphenol A (BPA) is one of the chemicals that is firmly accompanied by hippocampal neuronal injury. As oxidative stress appears to be a major contributor to neurotoxicity induced by BPA, antioxidants with remarkable neuroprotective effects can play a valuable protective role. Around the world, ( −)-epigallocatechin-3-gallate (EGCG) was one of the most popular antioxidants that could exert a beneficial neuroprotective role. Here, we examined the potential efficiency of EGCG against neurotoxicity induced by BPA in the hippocampal CA3 region of the rat model. This study revealed that EGCG was unable to abrogate the significant decrease in circulating adiponectin level and hippocampal superoxide dismutase activity as well as an increase in hippocampal levels of nitric oxide and malondialdehyde. Notably, EGCG failed to antagonize the oxidative inhibitory effect of BPA on hippocampal neurotransmission and its associated cognitive deficits. In addition, the histopathological examination with immunohistochemical detection of caspase-3 and NF-kB/p65 emphasized that EGCG failed to protect hippocampal CA3 neurons from apoptotic and necrotic effects induced by BPA. Our study revealed that EGCG showed no protective role against the neurotoxic effect caused by BPA, which may be attributed to its failure to counteract the BPA-induced oxidative stress in vivo. The controversial effect is probably related to EGCG’s ability to impede BPA glucuronidation and thus, its detoxification. That inference requires further additional experimental and clinical studies.

**Graphical abstract:**

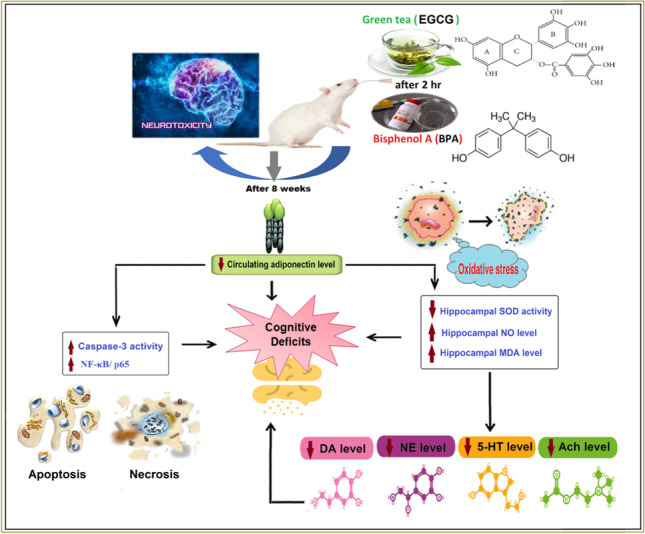

## Introduction

Bisphenol A (BPA; 4, 40-isopropylidenediphenol) is a widely used synthetic substance that has been found in a variety of everyday consumer items, including polycarbonate plastics and epoxy resins (Li and Suh 2019). Within BPA-containing containers, aging, heating, UV exposure, and changing pH will result in a process known as “leaching,” in which BPA polymers are broken apart and easily leak into food and drink contents (vom Saal and Welshons 2014). Because BPA is continuously released around the world, it can easily infiltrate the environment and then find its way into our bodies, causing a variety of negative health effects such as reproductive and developmental toxicity, metabolic disorders, immunotoxicity, neurobehavioral effects, and neurotoxicity (Bilal et al. 2019 and Li and Suh 2019).

Given that BPA exposure through food is the most dangerous of all the ways (Almeida et al. 2018), regulatory organizations such as the United States Environmental Protection Agency (USEPA) had to establish a reference safe dosage (RfD) for chronic oral BPA intake, which was established at 50 mg/kg/day. Several experimental investigations, however, have shown that even low doses of BPA, such as 5 mg BPA/kg/day for two weeks and 10 mg BPA/kg/day for six weeks, or eight weeks, can cause hippocampal cell damage (Kobayashi et al. 2020; Sadek et al. 2018).

The neurotoxic effect of BPA is supposed to arise from its antiestrogenic properties and accompanying overproduction of reactive oxygen and nitrogen species (ROS/RNS) (Chen et al. 2017), which was detected to inversely correlated with the serum adiponectin level (Li and Shen 2019). Through a mechanism involving the activation of the adenosine monophosphate-activated protein kinase (AMPK) signaling pathway, adiponectin was found to have a neuroprotective impact by decreasing ROS production and increasing eNOS activity (Rashtiani et al. 2021).

Simultaneously, multiple investigations highlighted the significance of free radicals in decreasing adiponectin production in adipocytes, affecting its secretion, and thereby interfering with its key role in preventing and healing neuronal apoptosis and necrosis (Bloemer et al. 2018). It is also worth noting that oxidative stress can impede the action of numerous enzymes involved in the biosynthesis of central neurotransmitters implicated in cognitive health, such as biogenic amines (dopamine (DA), norepinephrine (NE), and serotonin (5-HT)) and acetylcholine (Ach) (Haider et al. 2014).

Raising the prospect that an increase in oxidative stress is the primary cause of hippocampal neuronal injury and neurotoxicity associated with BPA, several recent studies have focused on the protective impact of various antioxidant agents against the oxidative damages caused by BPA (Amjad et al. 2020; El Morsy and Ahmed 2020; Mohammed et al. 2020). Likewise, there is a global trend toward the intake of natural plant-derived antioxidants due to their effectiveness in health promotion and disease prevention, as well as their capacity to increase safety and consumer acceptability (Szymanska et al. 2018). Green tea (*Camellia sinensis*, Theaceae), one of the most popular natural antioxidants ingested globally, has attracted a lot of attention for its helpful neuroprotective effect against neurodegeneration and neuronal damage (Malar et al. 2020; Prasanth et al. 2019).

In this regard, the most abundant and efficient green tea catechin, (–)-epigallocatechin gallate (EGCG), has been shown to have both direct and indirect antioxidant activities (Nikoo et al. 2018). EGCG has the ability to directly scavenge ROS by creating more stable phenolic radicals. In addition, it can effectively decrease malondialdehyde (MDA) levels (an indicator of oxidative stress) and increase superoxide dismutase (SOD) activity (an estimation of antioxidant activity) (Yan et al. 2020).

Despite the extensive research, there is no conclusive proof of the in vivo neuroprotective role of EGCG against oxidative stress and associated hippocampal neuronal damage caused by BPA. Hence, the effectiveness of EGCG as a powerful antioxidant and neuroprotective agent on the hippocampus of rats subjected to BPA is urgently needed.

## Materials and methods

### Animals

Specific-pathogen-free adult white male Wistar rats (weight range, 150–180 g) were obtained from the Experimental Animals Production unit of VACSERA, Giza, Egypt and housed under standard laboratory conditions for one week prior to the experiment for acclimatization. Rats were allowed for free access to standard diet and water ad libitum and maintained on a 12 h light/dark photoperiods.

### Chemicals and kits

BPA (> 99%, CAS: 80-05-7), corn oil (CAS: 8001-30-7), EGCG (> 95%, CAS: 989-51-5), Dulbecco’s phosphate-buffered saline (DPBS) (Cat No: D8537), fetal bovine serum (FBS) (Cat No: F9665), 0.4% trypan blue solution (Cat No: T8154), and colorimetric assay kit for caspase-3 (Cat No: CASP-3-C) were purchased from Sigma-Aldrich Co. Inc. (St. Louis, MO, USA). Bio diagnostic kits for determining the levels of both nitric oxide (NO) (Cat No: NO2533), MDA (Cat No: MD2528), and the activity of SOD (Cat No: SD2521) were obtained from Bio-Diagnostics Co. (Dokki, Giza, Egypt). Rat ELISA Kit for adiponectin (Cat No: EK1327) was obtained from Boster Biological Technology Co. (Ltd., USA). Colorimetric assay kit for measuring acetylcholine (Ach) concentration and acetylcholinesterase (AChE) activity was obtained from BioAssay Systems (Hayward, USA) and BioVision Inc. (Milpitas, USA), respectively. Rabbit polyclonal anti-NF-κB/p65 antibody (Cat No: RB-1638-R7) was purchased from Lab Vision (USA). Rabbit monoclonal anti-caspase-3 antibody (Cat No: E87-ab32351) was purchased from Abcam (USA). Goat anti-rabbit immunoglobulin from Biocare Medical (USA) and Gibco^®^ 0.25% Trypsin-EDTA Solution (TE) (Cat No: 25200056) from Thermo Fisher Scientific Inc. (USA) was used. All other chemicals used were of analytical grade and available commercially.

### BPA solution preparation

Bisphenol A solution was prepared freshly every day by dissolving 40 mg of bisphenol A in 1 ml of ethanol/corn oil (1:9 vol/vol) to produce a concentration of 40 mg BPA/ml (Gayrard et al. 2013).

### EGCG solution preparation

The solution was daily prepared in the early morning by dissolving 5 mg of EGCG in 1 ml of sterile distilled water.

### Experimental protocol and treatment

Eighty-four Wistar rats were randomly divided into four groups; each group included 21 rats: (I) 6.25 ml of ethanol/corn oil/kg/day; corn oil (Co) group; (II) 10 mg EGCG/kg/day, EGCG group (Biasibetti et al. 2013); (III) 250 mg/kg/day; BPA group (Yıldız and Barlas 2013); (IV) 10 mg EGCG/kg/day 2 h before 250 mg BPA/kg/day; EGCG + BPA group (Ullmann et al. 2003). Animals received all administrations once every day via oral gavage (PO) for eight weeks. Rats from each group were weighed at the start of the experiment and every 2 weeks. Twenty-four hours after the end of the experiment, each rat group was randomly divided into two denominations: the first one consisted of 14 rats and was used for biochemical analysis and histopathological investigation; while the second denomination consisted of seven rats and was used for cognitive-behavioral analysis with a delay interval of 48 h in-between the test and another.

### Blood sample preparation

All rats, except those used for cognitive-behavioral analysis, were euthanized via cervical dislocation. Then, blood samples were collected, allowed to clot in a serum separator tube for about 30 min at room temperature, and centrifuged at 1000 × *g* for 15 min.

### Hippocampal tissue preparation

After blood sampling, the brain was carefully dissected out. For the hippocampus to be isolated easily, all other tissues along the convex outer side of the hemisphere as well as the meninges surrounding the hippocampus were removed, and then, hippocampi were ready to be isolated easily (Seibenhener and Wooten 2012). After isolation, all hippocampal samples were divided into two parts; one part was kept frozen at −20 °C until used for determining biochemical parameters, while another was fixed in 10% neutral buffered formaldehyde (pH 7.4) until used for assessing histopathology and immunohistochemistry.

### Serum biochemistry

The separated sera were used for determining the concentration of circulating adiponectin in μg/ml based on a technology of standard sandwich enzyme-linked immunosorbent assay (ELISA) and according to the manufacturer’s instructions.

### Hippocampal tissue biochemistry

#### Oxidant/antioxidant assay

The measurement of hippocampal NO, MDA concentrations, and SOD activity was performed using colorimetric assay kits based on the spectrophotometric method at 540 nm, 534 nm, and 560 nm for NO, MDA, and SOD, respectively (Armstrong 1963; Ohkawa et al. 1979; Sun et al. 1988). Data were expressed in µmol/g tissue and nmol/g tissue for NO and MDA, respectively and in µmol/min/g tissue for SOD.

#### Neurotransmitters assay

The frozen hippocampal tissue samples were weighed as (0.2 g), homogenized in Hcl-butanol, and centrifuged for (10 min) to be used for further estimation of DA, NE, and 5-HT using fluorescence spectorophotofluorometer (Schimadzu, RF- 500, Japan) at 385 nm/485 nm, 320 nm/385 nm after (20 min), and 360 nm/470 nm for NE, DA, and 5-HT, respectively (Jacobowitz and Richardson 1978). The values were expressed as μg/g tissue. For ACh and AChE activity, they were estimated using colorimetric assay kits at 570 nm according to the instructions provided. The values were expressed as μg/g tissue and μmol/min/mg protein for ACh and AChE activity, respectively.

#### Histopathology and Immunohistochemistry

Following the isolation of all left hippocampi, the isolated samples were fixed in 10% neutral buffered formalin (pH 7.4) for 72 h, washed, dehydrated, embedded in paraffin wax, serially sectioned with a microtome at 3 μm thickness, and stained with hematoxylin and eosin (H&E) for histopathological investigation. Other sections (5 μm) were used for immunohistochemical detection of caspase-3 and NF-κB/p65 antibodies.

#### Immunohistochemical detection of caspase-3 and NF-κB/p65

Detection of caspase-3 and NF-κB was based on the peroxidase/anti peroxidase (APA) technique by using rabbit monoclonal anti-caspase-3 and polyclonal anti-NF-κB/p65 as primary antibodies, together with goat anti-rabbit immunoglobulin as secondary antibodies. For detection of caspase-3 antibody, sections were subjected to antigen retrieval by boiling in Tris-buffered saline solution (0.05 M, pH 7.6) for 5 min, cool down at room temperature for 20 min, and rinsed with phosphate-buffered saline (PBS) for 1 min. Endogenous peroxidase was inactivated by immersing sections in 3% hydrogen peroxide for 10 min followed by washing in PBS. Blocking was done by incubation with normal goat serum. Sections were incubated overnight with the primary antibodies in a humidity chamber at 4 °C, then washed in PBS. Sections were incubated with the secondary antibody for 60 min at room temperature (RT), washed in PBS, incubated with peroxidase/anti peroxidase solution for 10 min at RT, and then rinsed with PBS. To develop a color reaction, one drop of 3-30-diamino-benzidine-tetra-hydrochloride (DAB) chromogen was added to 2 ml of DAB substrate, mixed, and applied on tissues for 5–15 min. Finally, sections were counterstained with Mayer’s hematoxylin.

For detection of NF-κB/p65 subunit, the protocol was typical as mentioned before except subjecting the hippocampal sections to antigen retrieval by boiling in citrate buffer (10 mM citric acid, 0.05% Tween 20, pH 6.0) for 10 min. Leica DMLB microscopes and Leica EC3 digital cameras were used.

#### Morphometric analysis

A minimum of five different areas for each specimen was examined at × 40, and the mean number of positive cells was counted and expressed as an apoptotic index (%) and necrotic index (%); which was the percent of mean apoptotic cells demonstrating distinct cytoplasmic positive immunostaining signals for active caspase-3 and mean necrotic cells showing nuclear positive NF-kB/p65 immunostaining to total cells counted, respectively.

### Cognitive-behavioral performance tests

#### Morris water maze (MWM)

Spatial learning and memory for rodents were commonly examined by utilizing MWM, in which animals should search for the correct path to find the platform is hidden under the water surface (Morris 1984). All conditions including the task time, pool length, temperature, light, and rat number in each group were fixed for all groups. A rectangular glass tank (40 cm × 70 cm in diameter × 60 cm in height, maintained at 25 °C) was divided into four equal quadrants and then filled to about 30 cm deep with warm water. A platform was submerged 2 cm below the water surface and fixed in one quadrant (El Tabaa et al. 2017). Firstly, rats were oriented on the platform for 15 s to determine the specific particular corners of the tank. After orientation, they were trained once a day for four consecutive days to locate the platform in the clear water during the specified maximum swimming time (60 s). If the animal finds the platform before 60 s, it had passed and could be removed. If the rat failed, it was allowed an additional 30 s and guided gently to locate it. On the fifth day, rats were allowed to swim freely for 1 min without a platform. Then, the same platform was being hidden in cloudy water and each rat was located facing the tank wall and in the center of other three quadrants that containing the hidden platform. Rats were tested four times a day for four consecutive days to find the hidden platform within 60 s relying on the spatial orientation to specific particular corners of the tank. Each rat was allowed to take a 15-min interval in between for rest in the waiting cage. In the end, animals were towel-dried and returned back to their home cages. Rat cognitive performance during swimming could be expressed by the latency time (s), which defined as the time taken by a rat to find the hidden platform per day.

#### Y-Maze

This task evaluated the spontaneous alternation behavior which is a measure of the working memory depending on the use of a maze that was made of three identical arms (Maurice et al. 1994). Each arm was 45 x 30 cm, blocked off from the end except one arm, which was provided with a small opening at its end from which rats could escape out of the maze. Rats were allowed to explore the maze by randomly entering each arm searching for a way to escape from the maze. The ability of rats to alternate depended on the fact that animals knew well which arms had been visited and then, preferred to investigate a new arm rather than returning to a previously entered one till find the way. A number of alternations were measured, which was defined as the total number of individual arm entries into all three arms divided by the maximum possible alternations (Alternation = no. of entries into all three arms/max no. of entries).

#### Novel object recognition (NOR)

The task assessed the ability of rats to recognize a novel object in the environment if the rat exhibited brief exposures to the familiar objects for 1 day three consecutive 3-min trials with an intertrial delay of 1 min (familiarization phase). Yet, when one of the familiar objects was replaced by a novel one, the rat was allowed to discriminate the novel object from the familiar for 1 day three consecutive 3-min trials with an interval of 1 min (test phase) (Ennaceur 2010). Then, three rectangular carton arenas measuring (65 × 40 cm in diameter × 50 cm in height) with an open top and disposable paper floor were used. All their four walls were covered with white papers to provide the same place conditioning. An empty arena was used for (habituation phase). The variables measured were the global habituation index (GI %) that can be calculated by dividing the total time spent by the rat for exploring familiar objects during the familiarization phase to that spent in the test phase over 3 min multiplying by 100. Recognition index (RI %) can be calculated by dividing time spent exploring the novel object by the total time spent exploring both familiar and novel objects in the test phase over 3 min multiplying by 100.

#### Statistical analysis

Statistical analysis was performed by using the statistical package SPSS version 14.0 for windows (SPSS Inc., Chicago, IL, 2005) and GraphPad Prism software version 5.0 (San Diego, USA, 2007). Data were expressed as mean ± SD for the indicated number of animals. Normality and homogeneity tests were checked for continuous variables. Statistical significance between means was analyzed by using one-way analysis of variance (ANOVA). For multiple comparisons, Tukey’s honestly significant difference (HSD) post-hoc test was used if necessary. For all experiments, *p* < 0.05 was considered statistically significant.

## Results

### EGCG pre-treatment and BPA exposure disrupted the oxidant/antioxidant balance in rat’ hippocampi

**Fig. 1:**
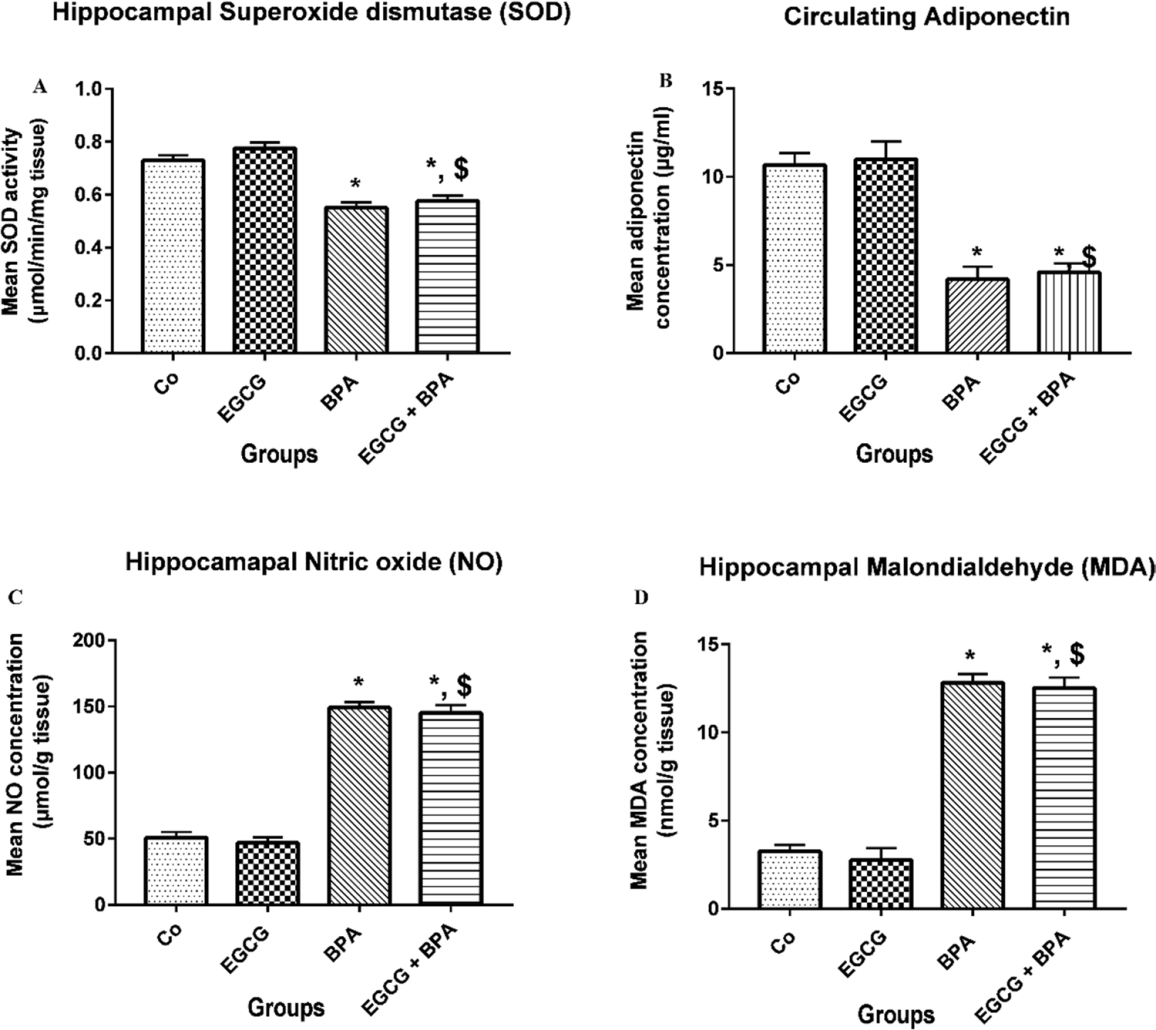
Effect of EGCG pre-treatment and BPA exposure on the oxidant/antioxidant balance in rats’ hippocampi. **A** SOD activity (µmol /min/g tissue), **B** circulating adiponectin (µg/ml), **C** NO concentration (µmol/g tissue), and **D** MDA concentration (nmol/g tissue). Rats were treated with corn oil (0.6 ml/kg/day, P.O.; Co), ( −)-epigallocatechin-3-gallate (10 mg EGCG/kg, P.O.; EGCG), bisphenol A (25 mg/kg, P.O.; BPA), and EGCG 2 h before BPA (EGCG + BPA) once every day for eight weeks. Data are presented as mean ± SD; One-way ANOVA followed by Tukey’s honestly significant difference (HSD) post-hoc; *n* = 7 per group. ^*, $^ Different symbols indicate significant difference (*p* < 0.05) vs. Co group or EGCG group, respectively

Figure 1 shows a significant decrease (*p* < 0.001) in hippocampal SOD activity and circulating adiponectin concentration, with a significant increase (*p* < 0.001) in the hippocampal concentrations of NO and MDA in rats pre-treated with EGCG 2 h before BPA exposure versus either Co group or EGCG group. At the same time, no changes were detected as compared to the BPA group.

### EGCG pre-treatment and BPA exposure decreased mean body weight

**Fig. 2:**
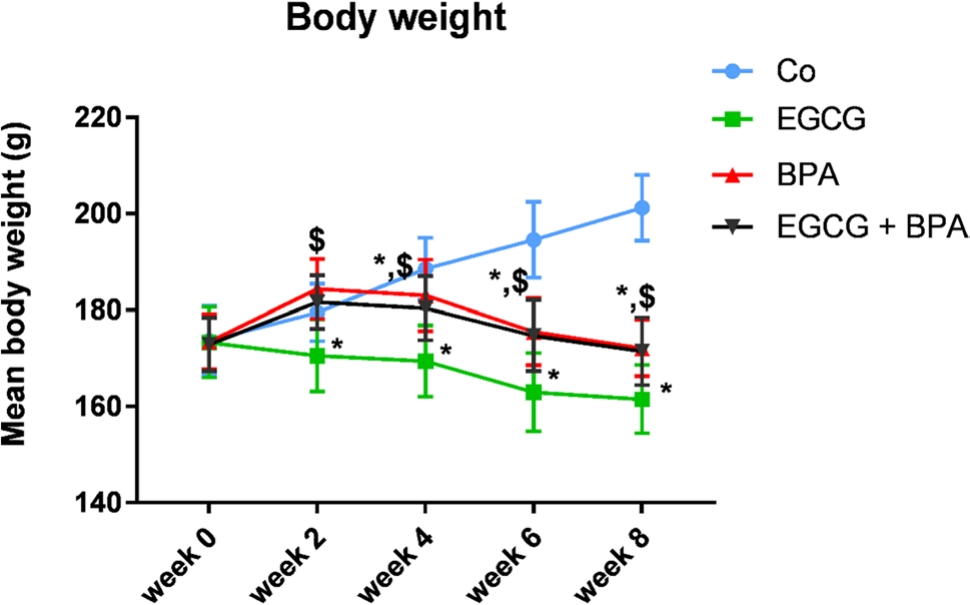
Effect of EGCG pre-treatment and BPA exposure on rat’s body weight. Rats were treated with corn oil (0.6 ml/kg/day, P.O.; Co), ( −)-epigallocatechin-3-gallate (10 mg EGCG/kg, P.O.; EGCG), bisphenol A (25 mg/kg, P.O.; BPA), and EGCG 2 h before BPA (EGCG + BPA) once every day for eight weeks. Data are presented as mean ± SD; One-way ANOVA followed by Tukey’s honestly significant difference (HSD) post-hoc; *n* = 21 per group. ^*, $^ Different symbols indicate significant difference (*p* < 0.05) vs. Co group or EGCG group, respectively

Figure 2 declares that the individual weight of rats pretreated with EGCG 2 h before BPA exposure was significantly decreased (*p* < 0.001) as compared to Co group during 4-, 6-, and 8-weeks, but with significant increase versus EGCG group and without changes versus BPA group from the start of the experimental period till the end**.**

### EGCG pre-treatment and BPA exposure had an inhibitory effect on rats’ hippocampal neurotransmission

**Fig. 3:**
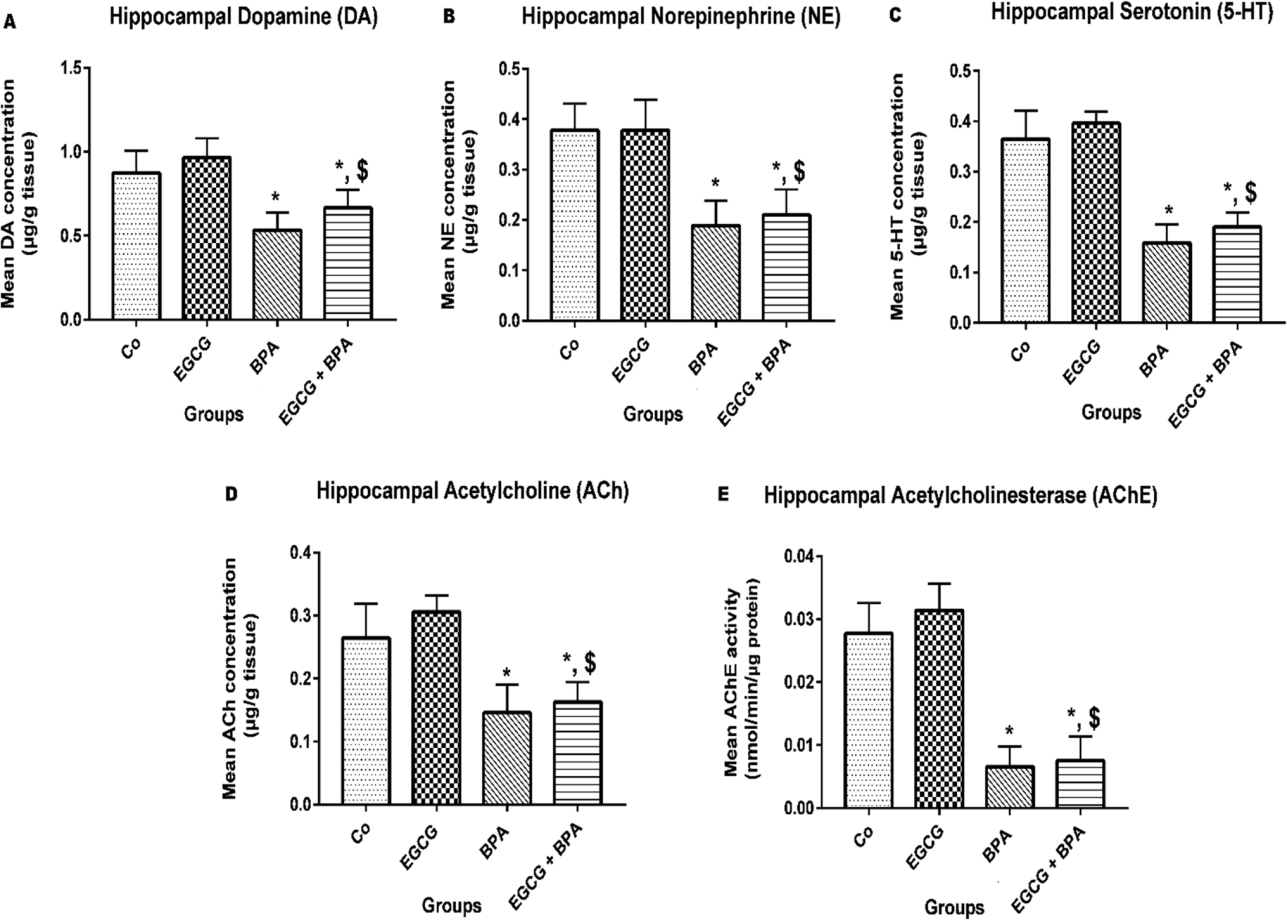
Effect of EGCG pre-treatment and BPA exposure on rats’ hippocampal neurotransmission. **A** Dopamine (DA) (µg/g tissue), **B** Norepinephrine (NE) (µg/g tissue), **C** Serotonin (5-HT) (µg/g tissue), **D** acetylcholine (Ach), and **E** acetylcholinesterase (AChE). Rats were treated with corn oil (0.6 ml/kg/day, P.O.; **Co**), ( −)-epigallocatechin-3-gallate (10 mg EGCG/kg, P.O.; EGCG), bisphenol A (25 mg/kg, P.O.; BPA), EGCG 2 h before BPA (EGCG + BPA) once every day for eight weeks. Data are presented as mean ± SD; One-way ANOVA followed by Tukey’s honestly significant difference (HSD) post-hoc; *n* = 7 per group. ^*, $^ Different symbols indicate significant difference (*p* < 0.05) vs. Co group or EGCG group, respectively

Figure 3 shows that there was a significant decrease (*p* < 0.001) in the hippocampal concentrations of DA, NE, 5-HT, and Ach as well as the hippocampal activity of AChE in rats pre-treated with EGCG for 2 h before BPA exposure as compared to either Co group or EGCG group. On the contrary, there was no change detected versus the BPA group.

### EGCG pre-treatment and BPA exposure induced histopathological alterations in rats’ hippocampal neurons of CA3 region

**Fig. 4:**
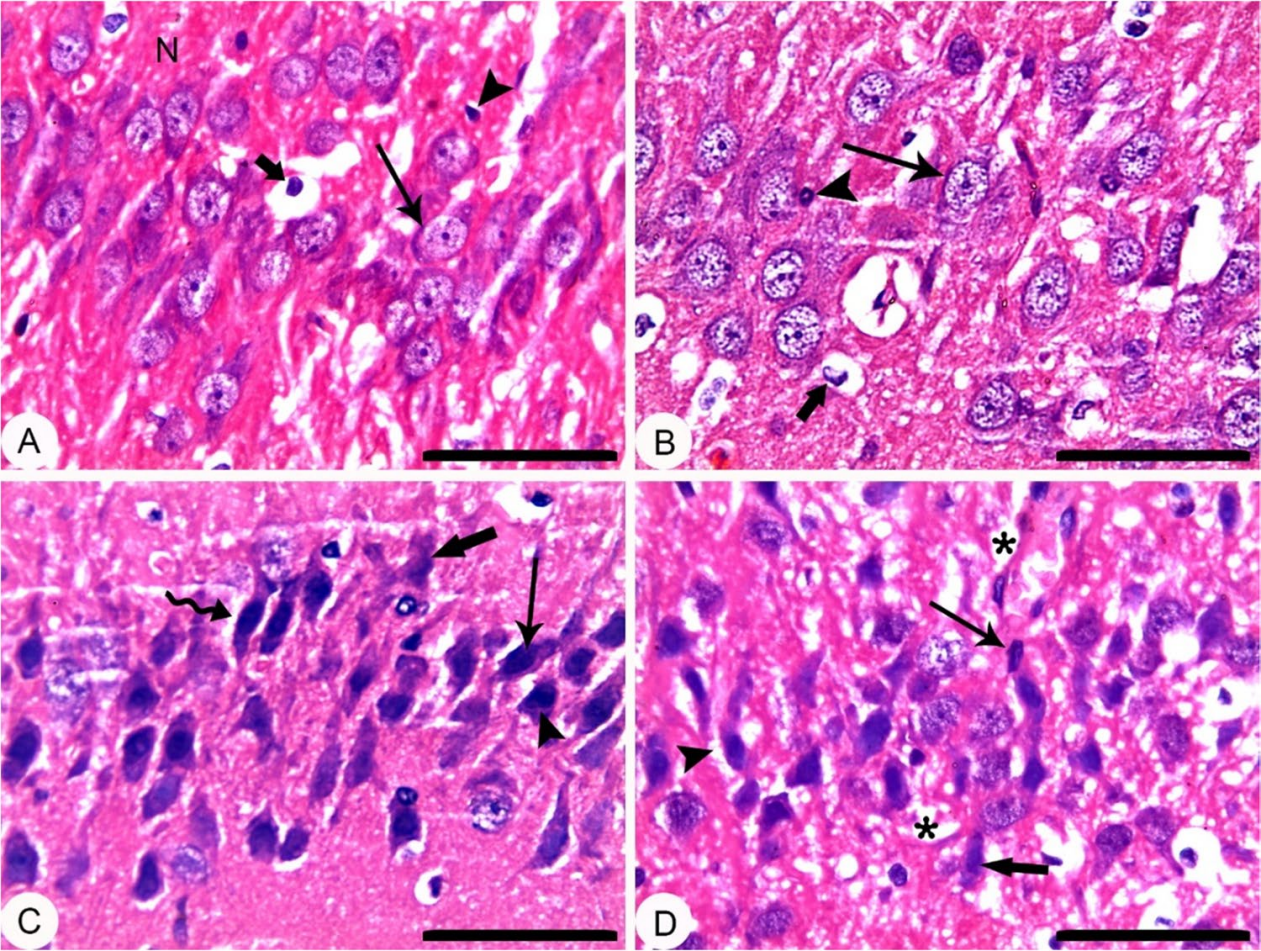
Hippocampus; CA3 region; rat; H&E stain; bar 50 µm: (**A**) Corn oil (Co), showing normal histological architecture. Pyramidal cells (thin arrow); astrocyte (thick arrow); Oligodendrocyte (arrowhead). (**B**) ( −)-epigallocatechin-3-gallate (EGCG), showing normal histological architecture. Pyramidal cells (thin arrow); astrocyte (thick arrow); Oligodendrocyte (arrowhead). (**C**) Bisphenol A (BPA); showing necrosis (thick arrow), apoptosis (thin arrow) in pyramidal cells, central chromatolysis of Nissl granule (arrowhead), and perineuronal edema (bended arrow). (**D**) EGCG + BPA; showing necrosis (thick arrow), apoptosis (thin arrow) in pyramidal cells, perineuronal edema (arrowhead), and vacuolation in neuropil (asterisk)

Figure 4 shows an abnormal pyramidal cells in hippocampi of rats pre-treated with EGCG 2 h before BPA exposure as compared to either the Co group or EGCG group. In addition, it revealed apoptosis and necrosis in pyramidal cells with perineuronal edema. Vacuolation in neuropil also appeared. No significant change was detected versus the BPA group.

### EGCG pre-treatment and BPA exposure confirmed apoptotic and necrotic effects in rat’ hippocampi

**Fig. 5:**
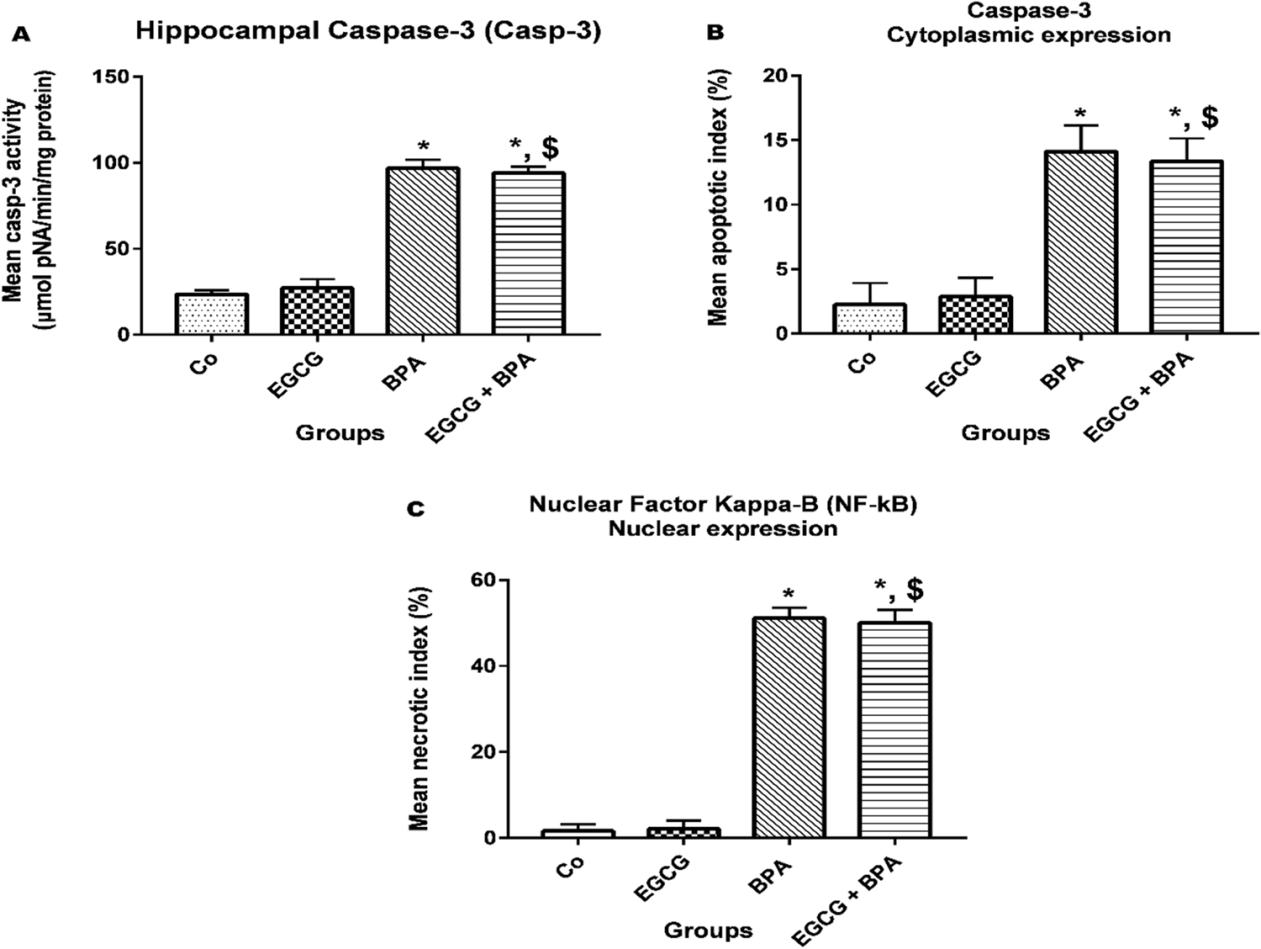
Apoptotic and necrotic effects of EGCG pre-treatment and BPA exposure on rats’ hippocampi. **A** Hippocampal Casp-3 activity (μmol pNA /min/mg protein), **B** mean apoptotic index (%), and **C** mean necrotic index serotonin (%). Rats were treated with corn oil (0.6 ml/kg/day, P.O.; Co), ( −)-epigallocatechin-3-gallate (10 mg EGCG/kg, P.O.; EGCG), bisphenol A (25 mg/kg, P.O.; BPA), EGCG 2 h before BPA (EGCG + BPA) once every day for eight weeks. Data are presented as mean ± SD; One-way ANOVA followed by Tukey’s honestly significant difference (HSD) post-hoc; *n* = 7 per group. *, ^$^ Different symbols indicate significant difference *(p* < 0.05) vs. Co group or EGCG group, respectively

**Fig. 6:**
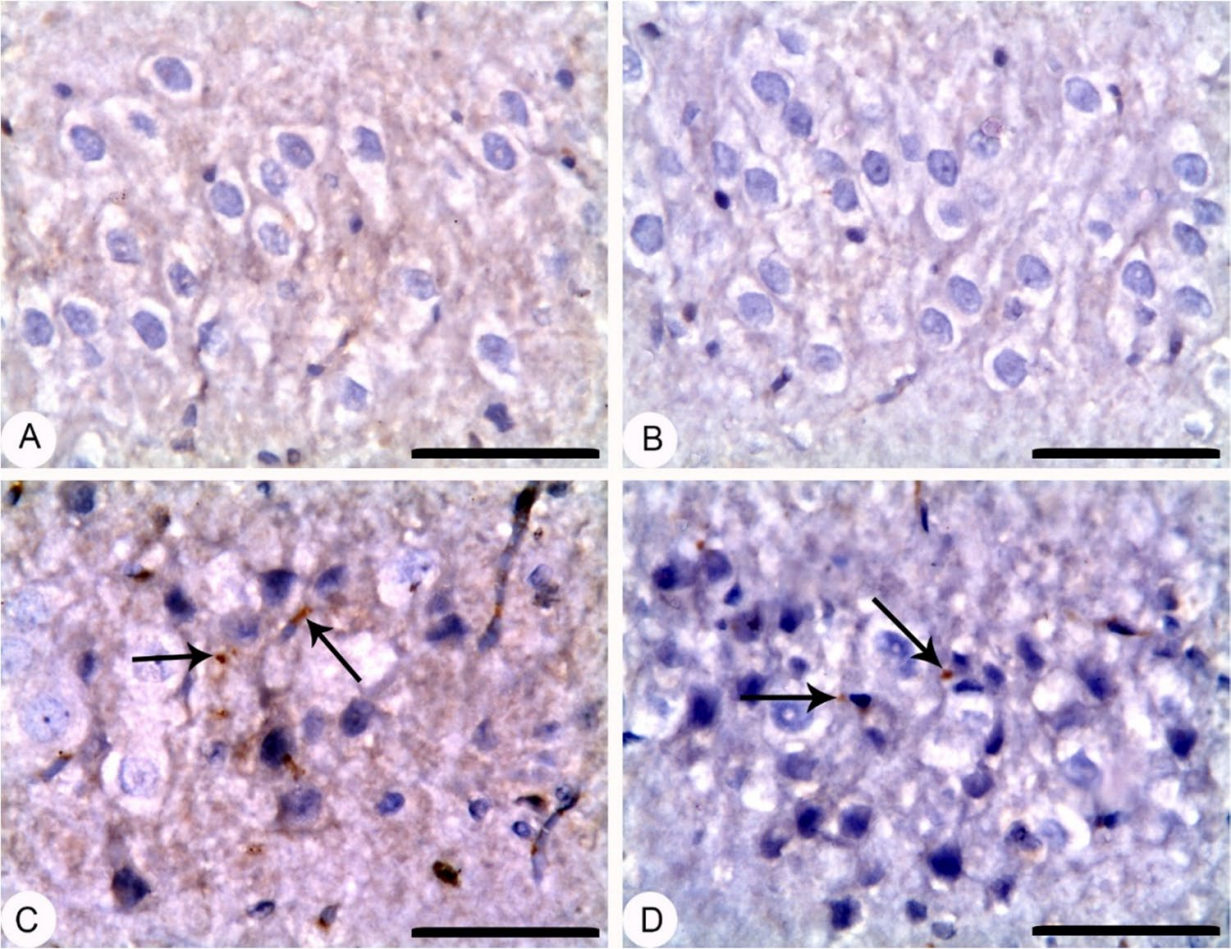
Hippocampus; CA3 region; rat; caspase-3 IHC; bar 50 µm: (**A**) Corn oil (Co) and (**B**) ( −)-epigallocatechin-3-gallate (EGCG) showed no signals in their hippocampal neurons. (**C**) Bisphenol A (BPA) and (**D**) EGCG + BPA showed an increase in the cytoplasmic expression of caspase-3 in the form of moderate positive brown immunostaining signals for active Casp-3 in their pyramidal hippocampal neurons of CA3 region as indicated by arrows

**Fig. 7:**
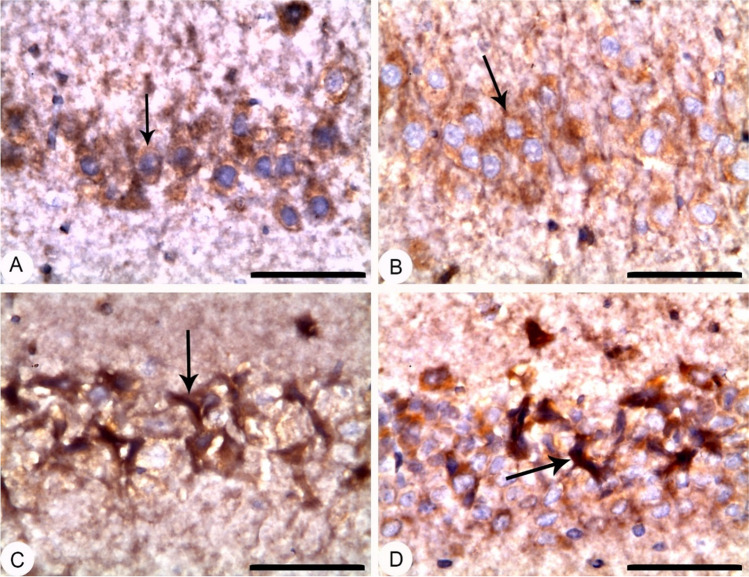
Hippocampus; CA3 region; rat; NF-κB/p65 IHC; bar 50 µm:** (A)** Corn oil **(Co**) and **(B)** ( −)-epigallocatechin-3-gallate (EGCG) showed no signals in their hippocampal neurons**.** (**C**) Bisphenol A (BPA) and (**D**) EGCG + BPA showed an increase in the nuclear expression of NF-кB in the form of strong positive nuclear staining for NF-κB/ p65 subunit in their pyramidal hippocampal neurons of CA3 region as indicated by arrows

Figure 5 declares that rats pre-treated with EGCG for 2 h before BPA exposure significantly increased (*p* < 0.001) hippocampal Casp-3 activity, mean apoptotic index, and mean necrotic index as compared to either Co group or EGCG group, but no changes were detected versus BPA group. At the same time, Figures 6 and 7 show a significant increase in the expression of Casp-3 and NF-кB in the form of moderate positive brown immunostaining signals for active caspase-3 and strong positive nuclear staining for NF-κB/ p65 subunit in the hippocampal neurons of the CA3 region, respectively.

### EGCG pre-treatment and BPA exposure had negative effects on cognitive-behavioral performance of rats

As shown in Table 1, rats pretreated with EGCG 2 h before BPA exposure showed a significant increase (*p* < 0.001) in the mean latency time during the MWM test with a significant decrease (*p* < 0.001) in the number of alternations and in GI and RI measured from Y-maze and NOR task, respectively, as compared to either Co group or EGCG group, without changes detected versus the BPA group.

**Table 1:** Negative effects of EGCG pre-treatment on cognitive-behavioral performance of rats exposed for BPA

Animal groups	(A) Latency time (sec)	(B) Number of alternations	(C) Global habituation index % (GI)	(D) Recognition index % (RI)
Co group	7.7 ± 2.63	2.39 ± 0.26	172.6 ± 7.85	0.83 ± 0.01
EGCG group	7.2 ± 2.39	2.40 ± 0.29	168.61 ± 7.91	0.82 ± 0.06
BPA group	29.1 ± 1.49 ^*, $^	1.3 ± 0.28 ^*, $^	77.31 ± 5.48 ^*, $^	0.36 ± 0.05 ^*, $^
EGCG + BPA group	27.8 ± 1.36^*, $^	1.39 ± 0.26 ^*, $^	78.61 ± 5.54 ^*, $^	0.38 ± 0.05 ^*, $^

**(A)** Latency time (s), the time taken to find the hidden platform per day; (B) Number of alternations, the total number of individual arm entries into all three arms divided by the maximum possible alternations; (C) Global habituation index (GI) (%), the percent of the time spent exploring the familiar object during familiarization phase to that in the test phase; and (D) Recognition Index (RI) (%), the time spent exploring the novel object to the total time spent exploring both objects during the test phase, were calculated for all groups. Rats were treated with corn oil (0.6 ml/kg/day, P.O.; Co), ( −)-epigallocatechin-3-gallate (10 mg EGCG/kg, P.O.; EGCG), bisphenol A (25 mg/kg, P.O.; BPA), EGCG 2 h before BPA (EGCG + BPA) once every day for eight weeks. Data are presented as mean ± SD; One-way ANOVA followed by Tukey’s honestly significant difference (HSD) post-hoc; *n* = 7 per group. ^*, $^ Different symbols indicate significant difference (*p* < 0.05) vs. Co group or EGCG group, respectively

## Discussion

Bisphenol A (BPA) is a dangerous environmental contaminant that has been implicated in the development of neurotoxicity (Santoro et al. 2019). Everyone has been forced to be exposed to the neurotoxic impact of BPA as a result of uncontrolled expansion in the manufacture and usage of BPA-containing commercial items (Mg et al. 2022). Various lines of evidence show that BPA can cause oxidative stress in the hippocampus, interfering with the synthesis and release of several central neurotransmitters as a result (Rebolledo-Solleiro et al. 2021). Jointly, antioxidants have received increased attention for their potential involvement in alleviating BPA-induced damage (Amjad et al. 2020).

Within this view, herbal medications that have been scientifically proven to be effective antioxidants and neuroprotective agents may have a protective impact against BPA-induced neurotoxicity. Green tea, which has been widely investigated for its protective effect against neurotoxicity and neuronal damage, was one of the most popular herbal antioxidants (Akbarialiabad et al. 2021). Furthermore, green tea has been demonstrated to significantly lower BPA-induced oxidative stress on erythrocytes in vitro and in silico studies (Suthar et al. 2014). Recently, it was shown that EGCG, the most abundant and potent green tea catechin, can reduce the neurotoxic effects of BPA in hippocampus neurons primary culture involving oxidative stress (Meng et al. 2021). Hence, it was imperious to assess the potential in vivo role of EGCG in protecting the rat hippocampus against BPA-induced neurotoxicity.

The findings of current study demonstrate that pre-treatment with EGCG 2 h before BPA exposure at a dose of (250 mg/kg/day) for eight weeks had no influence on the diminished level of circulating adiponectin that was strongly linked to BPA exposure. The results are consistent with (Haghighatdoost et al. 2017), who indicated that EGCG did not show any significant change in the level of circulating adiponectin.

Unlike to the prior studies ensuring the effective antioxidant action of EGCG, (Nikoo et al. 2018; Winiarska-Mieczan 2018; Yan et al. 2020), BPA-induced oxidative stress was not mitigated by EGCG pre-treatment, according to our findings. The failure of EGCG to restore the amount of circulating adiponectin, which has been shown to effectively control oxidative stress and its related cytotoxicity, might explain this outcome (Choubey et al. 2020).

The present study also showed that pre-treatment with EGCG 2 h before BPA resulted in a considerable reduction in body weight, which is paradoxical to the results of (Foula et al. 2020), who stated that there is an inverse relationship between the serum adiponectin level and body weight. Rather than being dependent on adiponectin levels, weight reduction may be triggered by a different mechanism. Previous research has suggested that the anti-obesity properties of EGCG are attributable to its ability to reduce food intake, disrupt lipid absorption, and decrease fat production (Huang et al. 2018).

Given the positive correlation between excessive free radical generation and high energy expenditure, it would seem natural that animals suffering from oxidative stress would lose their weight (Akohoue et al. 2007). Such a link can thus explain why rats given EGCG before BPA had lower body weights while having low circulating adiponectin levels.

Simultaneously, it is important to note that there was a link between body weight and cognition. Some studies have highlighted the relevance of weight reduction as a predictor of cognitive impairments (Xu et al. 2020). However, the precise processes are still unclear; indicating that body weight is not the only factor that might have a deleterious impact on brain function and structure.

Adiponectin has also been linked to the prevention and healing of neuronal damage, as well as the regulation of cognitive impairment. As a consequence, any drop in circulating adiponectin levels may be linked to the development of cognitive impairment (Bloemer et al. 2018). Meanwhile, multiple lines of evidence have pointed to the key involvement of biogenic amines (DA, NE, and 5-HT), as well as Ach, in alleviating cognitive impairments associated with neuronal damage (Štrac et al. 2016; Vazey and Aston-Jones 2012).

As well, our study declared that rats pretreated with EGCG before being exposed to BPA showed a decline in the levels of hippocampal biogenic amines (DA, NE, and 5-HT). As previously reported, the generation of oxidants inhibits the action of enzymes responsible for their primary biosynthesis (Castro et al. 2013; Elsworth et al. 2013). Given the negative effect of EGCG on BPA-induced oxidative stress, the generated ROS/RNS might explain the observed decline in DA, NE, and 5-HT levels in the hippocampus (Chen et al. 2017; Gassman 2017).

Furthermore, new research indicates that any decrease in neurotransmitter levels (DA, 5-HT, and Ach) is associated with erratic behavior and cognition (Kandeil et al. 2021). In the present study, pre-treatment with EGCG prior to BPA resulted in a low hippocampus Ach level, which coincided with a decrease in hippocampal AchE activity. Both activities appear to be paradoxical, because limiting AchE activity was known to be accompanied with an excess of Ach in the hippocampus (Hartmann et al. 1982).

The Ach-AChE cycle stated that ACh production is limited by the intracellular quantity of choline (a precursor for ACh formation) (Palmer Taylor and Joan Heller Brown 1999). Thus, inducing oxidative stress following BPA exposure in this study, which was not reduced even after EGCG therapy, may be the main cause for AchE enzyme damage, since ROS could alter its protein stability and subsequently, its affinity for Ach substrate (Afolabi et al. 2016).

That action will be definitely accompanied by a decrease in ACh biodegradation and thereby, the amount of choline available for uptake, which is considered as the rate-limiting step in ACh synthesis. Furthermore, producing more free radicals could also oxidize the reactive Cys thiols (Cys-S) of choline acetyl transferase (ChAT), which was reported to be an enzyme responsible for catalyzing the transfer of acetyl group into choline; resulting in a decrease in its activity and consequently, in Ach synthesis (Black and Rylett, 2011).

In view of the reality that clarifies a key role of hippocampus, especially CA3 pyramidal cells, in regulating the recognition and memory, it seemed obvious that any injury to hippocampal neurons would have a severe impact on cognitive performance (Barker and Warburton 2011). Because of its high quantity of polyunsaturated fatty acids (PUFAs) and a lack of antioxidant mechanisms, the hippocampus is particularly vulnerable to lipid peroxidation damage (de Freitas 2010).

In response to BPA exposure, caspase-3 can play a critical role in triggering apoptosis by cleaving essential cellular proteins, resulting in morphological abnormalities and cell death (Balci et al. 2020). The study noted that animals pretreated with EGCG before BPA exhibited both apoptosis and necrosis in the hippocampal neurons of the CA3 region, which can be elucidated by enhancing the activity of caspase-3 as a result of altering mitochondrial function by ROS/RNS. Otherwise, detection of active NF-κB/p65 subunit in hippocampal neurons would be a good proof for inducing necrosis during EGCG pre-treatment as a result of exposing neuronal cells into toxic oxidative stress markers including NO and MDA. These necrotic cells were established as potent inducers for activating NF-κB (Méndez-Armenta et al. 2014).

As worthily documented, EGCG can competitively inhibit the activity of one of the uridine glucuronosyltransferases (UGTs) enzymes; namely UGT1A1 (Gufford et al. 2014; Mohamed et al. 2010). The enzyme which may be involved in both intestinal and hepatic detoxification of BPA; minimizing its harmful toxic effects, in addition to UGT2B1 (the enzyme responsible for BPA metabolic pathway in rat liver) (Trdan Lušin et al. 2012; Yokota et al. 1999). Regrettably, rat UGT2B1 has been reported to be identical to human UGT2B17, which is likewise inhibited by EGCG (Jenkinson et al. 2012)*.* It is probable that the inhibitory impact of EGCG on BPA metabolism by both UGT1A1 and UGT2B1 enzymes is another plausible reason for inability of EGCG to alleviate BPA-induced oxidative stress and neuronal damage. Because limiting BPA metabolism would result in a rise in its free hazardous level in the bloodstream, it will certainly augment the risk of its entry into the hippocampus which itself lacks for UGTs isoforms required for BPA detoxification (Ouzzine et al. 2014).

**Fig. 8:**
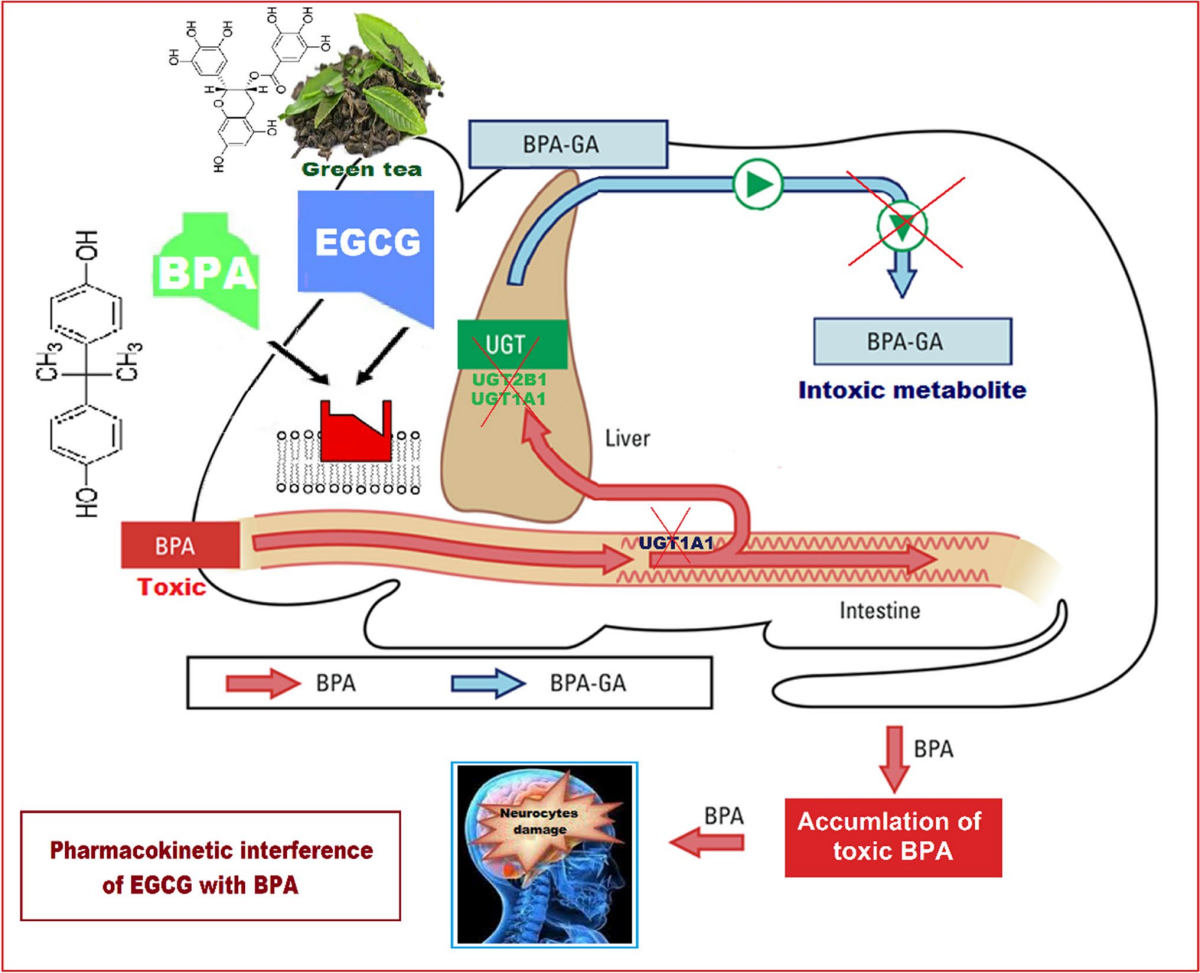
An expected mechanism for the negative role of ( −)-epigallocatechin-3-gallate (EGCG) against bisphenol A (BPA)-induced neurotoxicity in rats’ hippocampal neurons of CA3 region

Collectively, our data revealed that EGCG showed no effect on the neurotoxic effect of BPA in rats. EGCG pre-treatment did not alleviate the oxidative stress caused by BPA exposure nor did it counteract its detrimental effect on the survival, transmission, and function of hippocampal neurons, and hence on learning and memory functions. This unexpected impact might be attributable either to the negative role of EGCG towards BPA-associated low adiponectin levels, or to its ability to compete with intestinal and hepatic UGTs enzymes, hence preventing BPA metabolism and detoxification (Fig. 8).

Some limitations were identified in our study, such as the possibility that the BPA dosage employed was fairly high and thereby, hampered the protective effect of EGCG. Likewise, EGCG itself may be to blame for impairing the efficiency of antioxidant enzymes, especially when the treatment period is relatively long (8 weeks). Moreover, activities of the UGTs isoforms implicated in BPA detoxification have to be assessed in order to ensure the proposed inhibitory effect of EGCG. Hence, these might, at least in part, explain the in vivo neurotoxic effect in response to EGCG pre-treatment and BPA exposure, but future research will be necessary to confirm the proposed hypothesis clarifying the main reasons for the negative efficacy of EGCG against BPA-induced neurotoxicity in vivo.

## Data Availability

All datasets generated/analyzed during the present study are available.

## Abbreviations

BPA: Bisphenol A
EGCG: ( −)-Epigallocatechin-3-gallate
SOD: Superoxide dismutase
NO: Nitric oxide
MDA: Malondialdehyde
DA: Dopamine
NE: Norepinephrine
5-HT: Serotonin
ACh: Acetylcholine
AChE: Acetylcholinesterase
Casp-3: Caspase-3,;
NF-κB: Nuclear factor kappa-B
UGTs: Uridine glucuronosyltransferases
